# Mobility of the Native *Bacillus subtilis* Conjugative Plasmid pLS20 Is Regulated by Intercellular Signaling

**DOI:** 10.1371/journal.pgen.1003892

**Published:** 2013-10-31

**Authors:** Praveen K. Singh, Gayetri Ramachandran, Ricardo Ramos-Ruiz, Ramón Peiró-Pastor, David Abia, Ling J. Wu, Wilfried J. J. Meijer

**Affiliations:** 1 Centro de Biología Molecular “Severo Ochoa” (CSIC-UAM), Instituto de Biología Molecular “Eladio Viñuela” (CSIC), Universidad Autónoma, Canto Blanco, Madrid, Spain; 2 Parque Científico Madrid, Unidad de Genómica, Madrid, Spain; 3 Centre for Bacterial Cell Biology, Institute for Cell and Molecular Biosciences, Newcastle University, Newcastle Upon Tyne, United Kingdom; Universidad de Sevilla, Spain

## Abstract

Horizontal gene transfer mediated by plasmid conjugation plays a significant role in the evolution of bacterial species, as well as in the dissemination of antibiotic resistance and pathogenicity determinants. Characterization of their regulation is important for gaining insights into these features. Relatively little is known about how conjugation of Gram-positive plasmids is regulated. We have characterized conjugation of the native *Bacillus subtilis* plasmid pLS20. Contrary to the enterococcal plasmids, conjugation of pLS20 is not activated by recipient-produced pheromones but by pLS20-encoded proteins that regulate expression of the conjugation genes. We show that conjugation is kept in the default “OFF” state and identified the master repressor responsible for this. Activation of the conjugation genes requires relief of repression, which is mediated by an anti-repressor that belongs to the Rap family of proteins. Using both RNA sequencing methodology and genetic approaches, we have determined the regulatory effects of the repressor and anti-repressor on expression of the pLS20 genes. We also show that the activity of the anti-repressor is in turn regulated by an intercellular signaling peptide. Ultimately, this peptide dictates the timing of conjugation. The implications of this regulatory mechanism and comparison with other mobile systems are discussed.

## Author Summary

Bacteria evolve rapidly due to their short generation time and their ability to exchange genetic material, which can occur via different processes, collectively named Horizontal Gene Transfer (HGT). Most bacteria contain, besides a single chromosome, autonomously replicating units called plasmids. Many plasmids carry genes enabling them to be transferred into plasmid-free bacteria. This process, called conjugation, contributes significantly to HGT. Many plasmids also contain antibiotic resistance genes. Therefore, plasmid conjugation plays a major role in the spread of antibiotic resistance. Understanding the regulation of conjugation genes is essential for designing strategies to combat the spread of antibiotic resistance. We have studied the regulation of the native plasmid pLS20 from *Bacillus subtilis*. Besides being a soil bacterium, *B. subtilis* is a gut commensal in animals and humans. Here we unraveled the mechanisms controlling conjugation and found that pLS20 conjugation genes become activated when plasmid-free recipient cells are present. We have identified the repressor protein that keeps conjugation in an ‘OFF’ state, and an anti-repressor that activates conjugation. The activity of the anti-repressor is inhibited by a pLS20-encoded peptide that is secreted from the cell and can be absorbed by cells, after a secondary processing step. Ultimately, it is the signaling-peptide that dictates when conjugation genes become activated.

## Introduction

Horizontal Gene Transfer (HGT) plays a significant role not only in bacterial evolution but also in the spread of antibiotic resistance and pathogenicity determinants. The main mechanisms responsible for HGT are transformation mediated by natural competence, transduction, phage-related chromosomal islands (PRCI) and conjugation performed by plasmids or ICEs [1]–[4]. Conjugation is the process by which a DNA element is transferred from a donor cell to a recipient cell. Consequently, conjugation requires direct contact between the donor and the recipient cells. Often conjugative elements are present on plasmids, but they can also be found as mobile elements that are integrated in a bacterial chromosome. These latter forms are generally named integrative and conjugative elements (ICE).

The basics of the conjugation mechanism among plasmids are conserved. For a plasmid to be conjugative it requires a set of genes encoding proteins that (i) process the plasmid DNA into the form that can be transferred, which generally is single-stranded DNA, and (ii) generate a membrane-associated mating channel, called transferosome, through which the ssDNA is transported. The intercellular transferosome is a form of type IV secretion system. Generation of the ssDNA plasmidic form involves a relaxase, which forms a nucleoprotein complex called the relaxosome that introduces a site- and strand-specific nick within the origin of transfer (*oriT*). The relaxase remains covalently attached to the nicked DNA and the relaxasome is linked to the transferosome via the so-called coupling protein. Upon transfer of the ssDNA strand into the recipient cell through the transferosome, the attached relaxase directs recircularization of the ssDNA in the recipient cell.

Good understanding of the process of conjugation and its transcriptional regulation can provide insights into bacterial evolution. Such knowledge will also have important socio-economic, medical and biotechnological implications. For instance, it may provide valuable information to help control the explosive global spread of antibiotic resistance, and it may form the basis to construct tools to modify clinically or industrially important bacteria that are reluctant to genetic manipulation by other approaches. The process of conjugation and its transcriptional regulation has been studied in considerable detail for various plasmids present in Gram-negative (Gram^−^) bacteria (for review see, [5]–[8]). However, comparatively little is known about conjugation systems on plasmids from Gram-positive (Gram^+^) bacteria, many of them industrially and medically important organisms, although interest in this field is increasing (for general review see, [7], [9]). The conjugation machineries of plasmids from some Gram^+^ bacteria have been studied in more depth. Examples of these are (i) the broad host-range plasmid pIP501, originally isolated from *Streptococcus agalactiae* [10], [11, and references therein], pCW3 of *Clostridium perfringens* [12], the *Staphylococcal aureus* plasmids pGO1 and pSK41 [13], [14], and the *Enterococcus faecalis* plasmids pAD1 and pCF10. For the latter plasmids their transcriptional regulation has also been studied (for review see, [15]–[17]). A characteristic feature of these latter plasmids is that conjugation is induced by pheromones that are produced by plasmid-free recipient cells.

*Bacillus subtilis* is one of best studied Gram^+^ bacteria [18], [19]. Although many natural isolates of *B. subtilis* harbor one or more plasmids [20], little is known about conjugation systems present on *B. subtilis* plasmids. The main reason for this is that most *B. subtilis* studies are based on a few plasmid-free strains. For this reason and the other reasons stated below, we chose to study the regulation of plasmid conjugation in *B. subtilis*. First, due to its ability to develop natural competence, its genome and resident plasmids are amenable to genetic manipulation [18], [19]. Second, *B. subtilis* is closely related to fastidious and pathogenic bacilli like *B. cereus* and *B. anthracis*, respectively, and more distantly related to the Gram+ pathogen *Listeria*. Third, being a soil-dwelling bacterium that is found all over the world, *B. subtilis* may interact with a plethora of other bacteria and can be an effective vehicle for the transit of genes to and from other bacteria. This may be further underlined by the fact that it has become clear in recent years that various *B. subtilis* strains are also gut commensals in animals and humans [21]. It is therefore not unlikely that *B. subtilis* plasmids play an important role in HGT at various levels and this warrants a better understanding of them. For our studies we chose the 65 kb *B. subtilis* plasmid pLS20, which has been identified originally in the *Bacillus subtilis natto* strain IFO3335 [22] and shown to be conjugative even in liquid medium [23], [24].

Earlier studies have determined the replication region of pLS20 [25], and showed that it uses a dedicated mechanism involving the actin-like Alp7A protein for its segregation [26]. In addition, we recently discovered that pLS20cat, a derivative of pLS20 carrying a chloramphenicol-resistance gene (Cm) [24], encodes a protein that suppresses the development of natural competence of its host [27]. Although it has been shown that the conjugation machinery is predominantly formed at the cell poles [28], little is known about the process of conjugation itself.

In this work we studied the transcriptional regulation of the pLS20 conjugation genes. We identified an Xre-type repressor as the main transcriptional repressor that keeps the pLS20 conjugation system in the default “OFF” state. We show that pLS20 conjugation is not activated by recipient pheromones. Instead, activation of conjugation is exerted by a plasmid-encoded anti-repressor that belongs to the family of Rap proteins; most other members of which are involved in regulation of developmental processes in *B. subtilis*. Moreover, we show that activation of the conjugation genes is ultimately controlled by a signaling peptide that regulates the activity of the anti-repressor. To our knowledge, such a regulatory circuitry mechanism has never been described before for plasmids.

## Results

### pLS20 conjugation is not activated by pheromones

**Figure 1:**
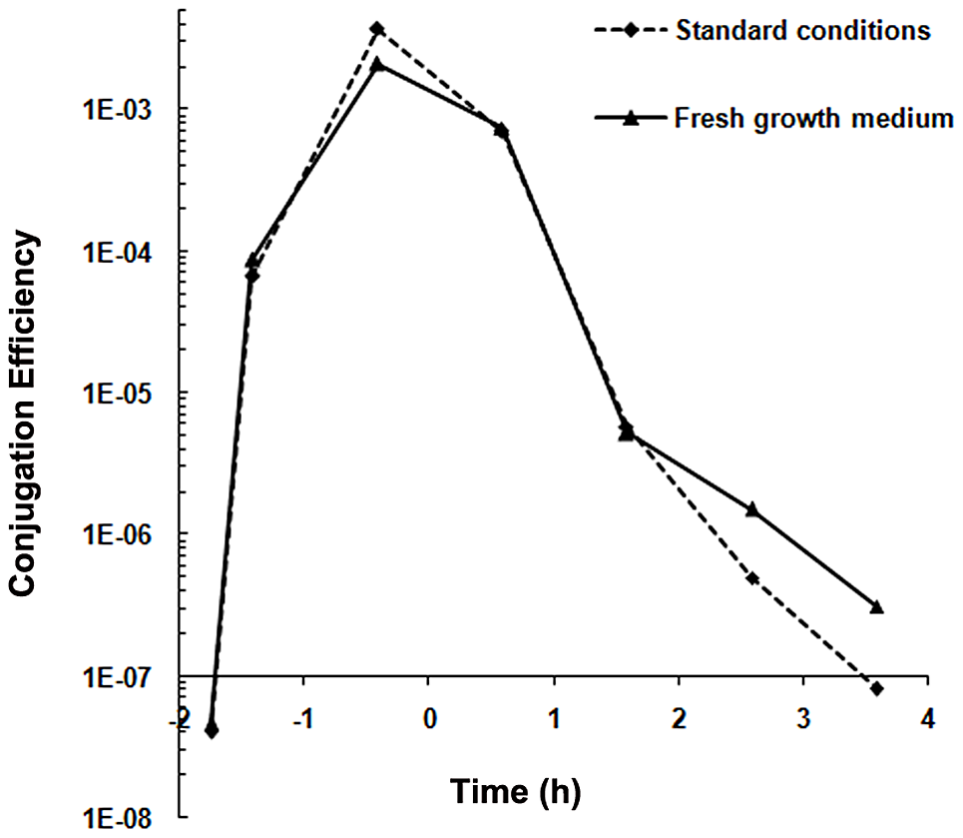
Conjugation kinetics of pLS20cat without and with replacing the recipient growth medium. Conjugation kinetics of pLS20cat was determined as described in Materials and Methods using strains PKS11 and PKS7 as donor and recipient strain, respectively. At each time point donor cells were mixed with recipient cells either directly (broken line) or after the recipient growth medium had been replaced with fresh LB medium (continuous line), and plated on selective agar plates after a 15 min mating period. t = 0 corresponds to the end of the exponential growth phase. Control experiments showed that the centrifugation step did not affect conjugation efficiency (not shown).

Conjugation systems present on Gram-positive *Enterococcus faecalis* plasmids are induced upon sensing a recipient-produced pheromone (for review see, [16]). To study whether the conjugation system of pLS20 is also induced by pheromones we determined conjugation efficiencies in liquid medium under different conditions using a Cm-labeled derivative of pLS20, pLS20cat [24]. Under the first condition, overnight grown cultures of donor (PKS11) and recipient (PKS7) cells were diluted and grown separately. At different times during growth, aliquots of the donor and recipient cells (∼1∶1 ratio) were mixed and their conjugation efficiencies were determined after a mating period of 15 min. The results presented in Figure 1 show that conjugation efficiencies increased during growth, reaching maximum levels near the end of the exponential growth phase, followed by a steep decrease in efficiencies at later times. The conjugation efficiency patterns obtained are similar to that published previously [24]. The observed increase in conjugation efficiency during the exponential growth phase might be due to accumulation of a conjugation activating signaling molecule produced by recipient cells. If this were the case, replacing the growth medium of the recipient cells with fresh medium before mixing with the donor cells should result in a reduction in conjugation efficiency. Figure 1 shows however that this treatment did not significantly affect conjugation efficiencies, strongly indicating that regulation of conjugation of pLS20 is fundamentally different from that of the enterococcal plasmids.

We then considered the possibility that recipient cells were specifically competent for conjugation during the mid to late exponential growth phase. However this was not the case either as similar levels of conjugation efficiencies were obtained regardless of the growth stage of the recipient cells (in the range of 10^−3^–10^−4^ transconjugants/donor). Altogether, these results indicate that the pLS20 conjugation system is not activated by recipient-produced signaling molecules. Instead, they support the view that under our standard conditions the conjugation system is continuously repressed except for a rather small window of time near the end of the exponential growth phase.

### Rco_LS20_, an Xre-type regulator protein encoded by pLS20 gene 27c, represses conjugation of pLS20

**Figure 2:**
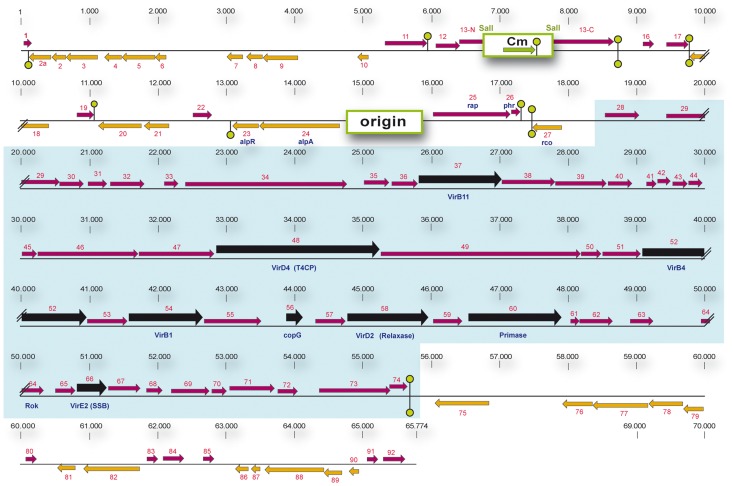
Genetic map of pLS20cat. (Putative) genes are numbered. Gene 1 corresponds to the homologue of gene 1 of the related *Bacillus pumilus* NRS576 plasmid p576 [37]. The positions and the lengths of the (putative) genes are indicated by arrows. Rightward and leftward oriented genes are indicated in purple and orange, respectively. Putative Rho-independent transcriptional terminators are indicated with green hairpin structures. The origin of replication region and the gene conferring resistance to chloramphenicol are labeled with green rectangles. The DNA region containing the chloramphenicol gene was cloned into the unique *Sal*I site located in pLS20 gene 13 [24]. The sequences flanking the Cm resistance cassette coding for the N- and C-terminal regions of gene 13 are labeled 13-N and 13-C, respectively. The putative conjugation operon encompassing genes 28 to 74, is highlighted by a blue background. Genes showing significant homology with genes reported to be involved in conjugation in other systems are shown in black. Recently, the complete pLS20cat sequence has been deposited by Itaya,M., *et al*. (Mitsuhiro Itaya Keio University, Japan) in public database under accession numbers NC_015148.1 and AB615352.1. pLS20cat gene 25, according to our nomenclature, corresponds to gene 001 of the deposited sequence. Due to differences in annotation we prefer to maintain our nomenclature.

The observation that efficient conjugation occurred only during a short time window raised the possibility that conjugation is kept in the default “OFF” state by a transcriptional repressor protein, and is switched on only in a certain period during growth when the repressor is inactivated. To identify a possible conjugation repressor gene we sequenced and annotated pLS20cat, and used this information to construct a genetic map of pLS20cat (Figure 2). The following features identified gene 27c as a possible candidate encoding a conjugation repressor. First, *in silico* analysis indicated that it encodes an Xre-type, transcriptional regulator with a Helix-Turn-Helix (HTH) domain in its N-terminal region (see Figure S1). Second, gene 27c is located immediately upstream of a divergently oriented putative conjugation operon spanning genes 28 to 74. Several of the genes in the 28 to 74 region are predicted to be homologues of essential conjugation genes present on other conjugative plasmids, and homologues of essential conjugation genes are not found outside this region of pLS20cat (see Figure 2). Table S1 gives an overview of the comparative analysis of genes in this region that includes details on the putative translation start sites.

To test whether gene 27c indeed encodes a repressor of the conjugation genes we studied the effect of ectopic expression of gene 27c on pLS20cat conjugation. For this, we constructed strain PKS14 that harbors plasmid pLS20cat and contains an ectopic copy of gene 27c under the control of the IPTG-inducible P_spank_ promoter at the chromosomal *amyE* locus. Conjugation efficiencies for pLS20cat were determined when PKS14 donor cells were grown in the presence or absence of IPTG. Since maximum conjugation levels occur near the end of the exponential growth phase (see above), we first determined conjugation efficiencies of pLS20cat during this phase. As a control, conjugation efficiencies of pLS20cat were determined in the wild type background (strain PKS11). The results presented in Table 1 show that ectopic expression of pLS20cat gene 27c resulted in a dramatic decrease (>50,000 fold) in pLS20cat conjugation efficiency, supporting the view that it encodes a repressor of conjugation. In the absence of inducer, strain PKS14 showed a small but noticeable decrease in conjugation efficiency (25 to 30-fold) compared to that of strain PKS11 (pLS20cat in the wild type background). This was probably due to the leakiness of the P_spank_ promoter. Based on these results and those presented below we denominated gene 27c of pLS20 *rco_LS20_* (repressor of conjugation).

**Table 1:** pLS20 gene 27c (*rco_LS20_*) encodes a repressor of conjugation.

Strain	genotype	Plasmid	IPTG (1 mM)	Conjugation efficiency *
PKS11	168 (wt)	pLS20cat	−	5.6 10^−3^
			+	3.8 10^−3^
PKS14	*168, amyE*::P_spank-_ *rco_LS20_*	pLS20cat	−	1.6 10^−4^
			+	<1 10^−8^
PKS86	168, *amyE*::P_spank-_ *rco_LS20_*	pLS20rco	−	5.7×10^−2^
			+	<1×10^−8^

* : Conjugation efficiencies are calculated as transconjugants/donor. Conjugation efficiencies are the mean value of at least three independent experiments.

**Figure 3:**
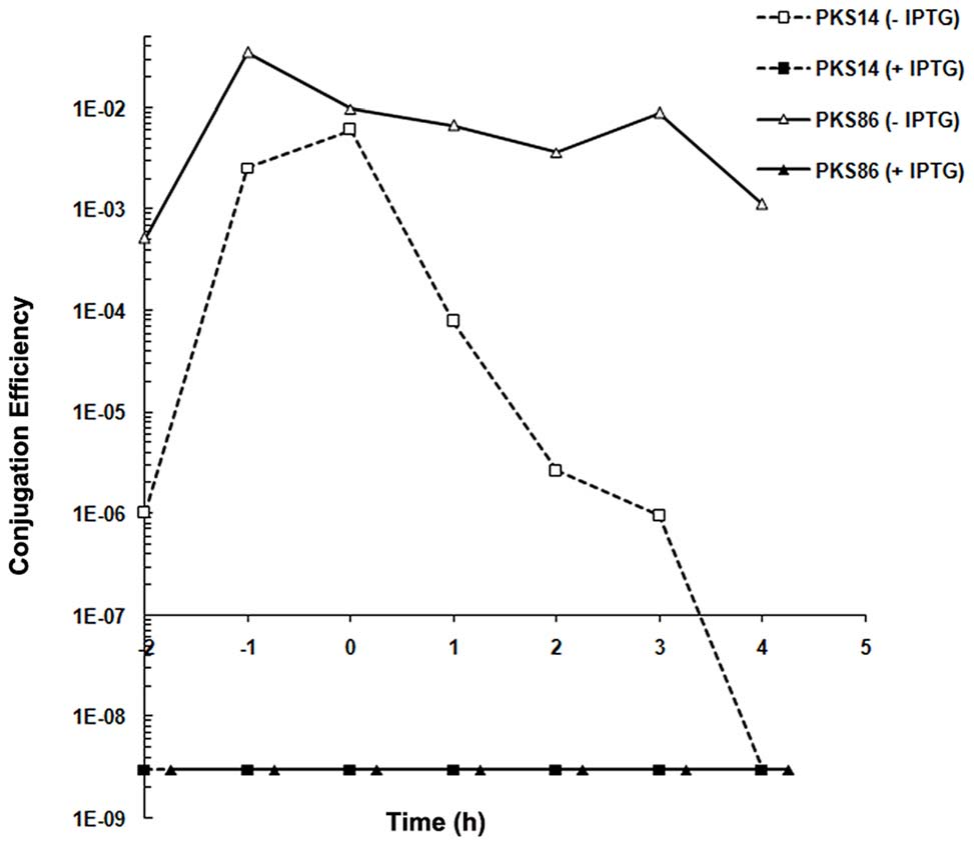
Gene 27c of pLS20cat encodes a repressor of conjugation. Conjugation kinetics of pLS20cat and pLS20rco were determined with and without ectopic expression of pLS20 gene 27c as described in Materials and Methods. PKS7 was used as recipient strain. Donor strains PKS14 (pLS20cat) and PKS86 (pLS20rco) both contain an ectopic copy of pLS20cat gene 27c under the IPTG inducible P_spank_ promoter at the chromosomal *amy*E locus. t = 0 corresponds to the end of the exponential growth phase. The conjugation efficiencies obtained for strains PKS14 and PKS86 were below the detection level of 1×10^−8^ when grown in the presence of IPTG.

To test the function of gene 27c more directly, we constructed a derivative of pLS20cat, pLS20rco, in which gene 27c is deleted and replaced by a kanamycin marker. The expected constitutive de-repression of the conjugation operon in the absence of Rco in pLS20rco might pose a burden to the cell. Therefore, we introduced pLS20rco into strain PKS9 containing the P*_spank_*-*rco_LS20_* construct. The resulting strain, PKS86, was used to determine the kinetics of conjugation during growth. Strain PKS14 containing the wild type pLS20cat in the same background, was included as a control. When *rco_LS20_* gene was expressed ectopically, the conjugation levels of both pLS20cat and pLS20rco were below the detection level of 1×10^−8^ at all time points tested, confirming that Rco_LS20_ represses conjugation (Figure 3). Interestingly, in the absence of ectopic Rco_LS20_ expression conjugation efficiencies of pLS20rco differed in two aspects from that of pLS20cat. First, conjugation efficiencies were higher at all time points measured; and second, conjugation levels were high for a very broad window of time. Therefore, in the absence of a functional *rco_LS20_* gene conjugation was no longer inhibited, most likely because the conjugation genes were not repressed (Figure 3).

### Transcriptional analysis of pLS20cat genes by RNA-seq

**Figure 4:**
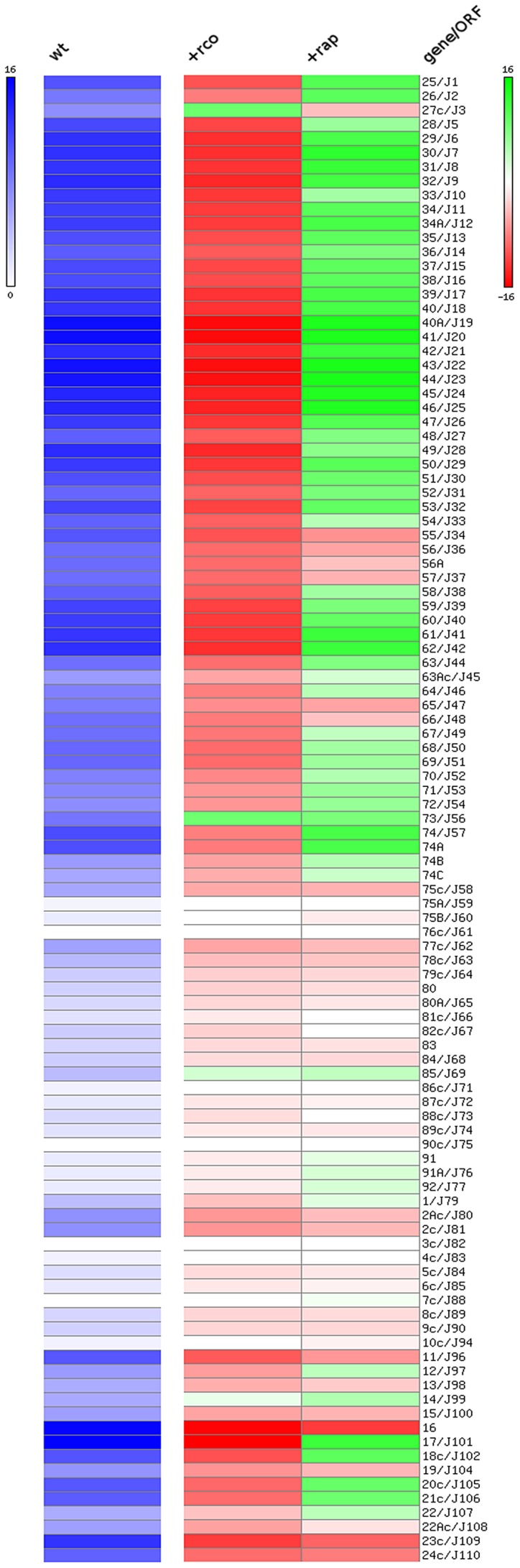
Heat map representation of the expression levels of the pLS20cat genes at late exponential phase under various conditions analyzed by RNAseq. Left lane (“wt”) shows the expression level of pLS20cat genes in the wild type strain background at late exponential phase when conjugation efficiency is at its maximum. Expression levels are presented on a log_2_ scale covering a range from 0 (white, lowest level) to 16 (blue, highest level). Middle (+Rco) and right (+Rap) lanes represent the effects of ectopic expression of Rco_LS20_ (middle lane) or Rap_LS20_ (right lane), respectively, on the expression of the pLS20cat genes. Differential expression levels are presented on a log_2_ scale covering a range of −16 to 16 using shades of red and green for repression and overexpression, respectively. White reflects no change in expression. Gene numbers according to our nomenclature and those deposited in database under accession number NC_015148.1 (preceded by “J”) are given on the right). “c” corresponds to leftward oriented genes.

Results presented above show that Rco_LS20_ suppresses conjugation. To establish whether Rco_LS20_ exerts its inhibitory effect on conjugation at the level of transcription and to identify genes that are under the control of Rco_LS20_, we performed RNAseq analysis to determine the expression pattern of all pLS20cat genes in a wild type background, and when grown in the presence of ectopic Rco_LS20_ expression. Thus, total RNA was isolated from late exponential phase cells of PKS11, and of PKS14 grown in the presence of IPTG. In parallel, total RNA was isolated from plasmid-free *B. subtilis* 168 cells grown under the same conditions to serve as a negative control. After processing, the RNA samples were used to generate cDNA libraries using a “directional RNA-seq” procedure that preserved information about a transcript's direction. The generated libraries were subjected to Illumina sequencing resulting in a total of about 56.5×10^6^ reads of 36-nt that passed the quality control settings. Of these, 1,596,385 reads mapped to the pLS20cat genome, and were used to calculate the apparent expression level of individual genes. A heat map representation of the expression levels of the pLS20cat genes when conjugation efficiencies were at their maximum is shown in the left lane of Figure 4. The middle lane in Figure 4 represents the effect of ectopic Rco_LS20_ production on the expression of the pLS20cat genes. Thus, increasing and decreasing RNA levels of individual genes are reflected by the intensity of green and red colors, respectively. The right lane (+rap) is explained further below. The additional expression of gene 27c encoding Rco_LS20_ from the ectopic locus is reflected by the green color of the corresponding rectangle. Importantly, the heat map shows significantly reduced RNA levels for genes 28 to 72, as well as gene 74, indicating that Rco_LS20_ is responsible for repressing these genes. Some other genes outside the region spanning 28–74 are also repressed under these conditions. Further analysis using quantitative RT-PCR confirmed these results (data not shown). At present, we do not know whether Rco_LS20_ represses these genes directly or indirectly, but the results clearly show that Rco_LS20_ represses genes 28 to 72 as well as other putative plasmid genes encoding proteins of unknown function, such as genes 11 and 16–21c.

### Rap_LS20_ is not involved in sporulation or competence but stimulates conjugation by counteracting Rco_LS20_-mediated repression

Located downstream of the repressor gene *rco_LS20_* in pLS20cat is a putative *rap-phr* cassette (genes 25–26); the genes which we name *rap_LS20_* and *phr_LS20_*, respectively (see Figure 2). The genome of *B. subtilis* contains eleven *rap* genes. The name *rap* refers to the activity of the founding member RapA shown to be a Regulator Aspartate Phosphatase [29]. The functions of Rap proteins are to interfere with developmental processes such as sporulation, competence development and production of degradative enzymes and antibiotics [29]–[35]. In addition, *rap* genes have been identified on rolling-circle and theta replicating plasmids from *B. subtilis* and on the *Bacillus anthracis* megaplasmid pXO1 [25], [36]–[38]. For those analyzed, plasmid-encoded *rap* genes also affect the production of extracellular proteases or sporulation [38]–[40]. Based on this, it seemed plausible that *rap_LS20_* too could play a role in sporulation and/or competence. To test this, we constructed strain GR20, which contains a copy of *rap_LS20_* at the chromosomal *amyE* locus under the control of the inducible P_spank_ promoter. Surprisingly though, overexpression of Rap_LS20_ did not significantly affect sporulation or competence (supplemental Table S2).

**Figure 5:**
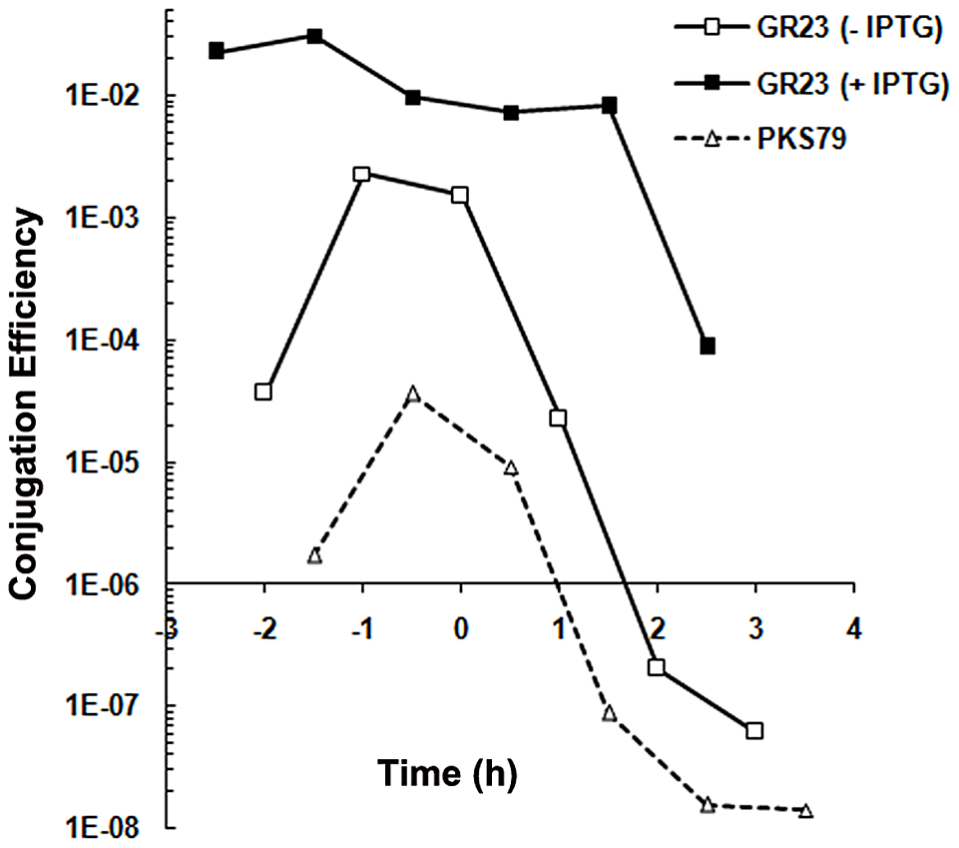
Rap_LS20_ stimulates conjugation. Conjugation kinetics of pLS20cat and pLS20rap were determined with and without ectopic expression of Rap_LS20_ as described in Materials and Methods. PKS7 was used as recipient strain. GR23 (pLS20cat) and PKS79 (pLS20rap) were used as donor strains. GR23 contains an ectopic copy of *rap_LS20_* under the control of the IPTG inducible P_spank_ promoter at the chromosomal *amy*E locus. t = 0 corresponds to the end of the exponential growth phase. Control experiments showed that overexpression of Rap_LS20_ in strain GR20 did not significantly affect growth (not shown).

The particular gene arrangement, being that the *rap*-*phr* cassette flanks *rco_LS20_*, stimulated us then to investigate the possibility that *rap_LS20_* could be involved in pLS20 conjugation. For this, we introduced pLS20cat into strain GR20 containing the inducible *rap*_LS20_ gene, and used the resulting strain GR23 to determine the kinetics of pLS20cat conjugation efficiencies in the absence and presence of ectopic Rap_LS20_ induction (Figure 5). Interestingly, ectopic expression of Rap_LS20_ stimulated conjugation. In fact, the kinetics of conjugation obtained under these conditions was similar to those obtained for pLS20rco, the derivative containing a deletion of gene *rco_LS20_* encoding the repressor of conjugation. Thus, in both cases, the maximum levels of conjugation increased and efficient conjugation occurred during a much broader time window. These results are a strong indication that Rap_LS20_ acts to counteract the Rco_LS20_-mediated repression of pLS20 conjugation.

The results of two additional approaches support this view. First, we determined the expression profile of pLS20cat genes in strain GR23 when *rap_LS20_* was expressed ectopically by RNAseq. A heat map representation of the results is presented in the right lane of Figure 4. Interestingly, almost all of the pLS20cat genes whose expression was repressed by Rco_LS20_ (middle lane, red rectangles), most noticeably genes 28 to 74 containing the predicted essential conjugation genes, were overexpressed when Rap_LS20_ was induced ectopically (right lane, green rectangles). Second, we deleted *rap_LS20_* from pLS20cat by replacing it with a Km marker, and then determined the conjugation kinetics of the resulting plasmid pLS20rap. Consistent with its role as a positive regulator, absence of *rap_LS20_* resulted in a severe reduction in conjugation efficiency (strain PKS79) (Figure 5). The combination of these results provides compelling evidence that Rap_LS20_ stimulates conjugation by relieving Rco_LS20_ mediated repression of the conjugation genes.

### RapI of *B. subtilis* ICE*Bs1* affects sporulation

The chromosomes of some *B. subtilis* strains contain a conjugative element, named ICE*Bs1* [41]. Transfer of this ICE has been shown to be activated by a member of the *rap* gene family, *rapI*, which is located within the ICE*Bs1* element [42]. Hence, both *rapI* and *rap_LS20_* play a role in the regulation of a conjugative element. Based on their similar function we expected that, like *rap_LS20_*, *rapI* would not affect sporulation. To test this prediction we constructed PKS139, an ICE-negative strain in which *rapI* is placed at *amyE* under the control of the IPTG inducible P_hyspank_ promoter, and used it to determine the efficiency of sporulation with and without induction of RapI. Surprisingly, sporulation efficiency dropped more than 200-fold when RapI was overexpressed, demonstrating that unlike Rap_LS20,_ RapI severely affected sporulation (supplemental Table S3).

### Phr*_LS20_ inhibits the activity of Rap_LS20_ and thereby determines the time window of efficient conjugation

**Figure 6:**
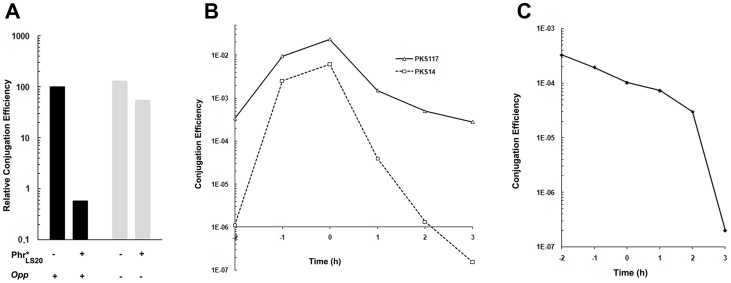
Phr*_LS20_ pentapeptide inhibits conjugation in an *opp* dependent manner. A. Effects of synthetic Phr* peptide on conjugation in the wild type and an *opp* deficient background. Conjugation efficiencies of pLS20cat were determined at late exponential growth phase using as recipient strain PKS7, and as donor either strain PKS11 (wild type, black bars) or PKS98 (*oppA*, grey bars). Diluted overnight grown cultures of donor cells were split in two, and Phr*_LS20_ pentapeptide was added to a final concentration of 6 µM to one of the cultures and equal volume of the peptide buffer to the other. B. Conjugation kinetics of pLS20cat and pLS20phr. Conjugation kinetics was determined as described in Materials and Methods using PKS7 as recipient strain and PKS14 (pLS20cat) or PKS117 (pLS20phr) as donor strains. t = 0 corresponds to the end of the exponential growth phase. Both donor strains contain an ectopic copy of *rco_LS20_* under the IPTG inducible P_spank_ promoter at the chromosomal *amy*E locus. Overnight cultures of donor cells were grown in the presence of 1 mM IPTG and diluted in fresh pre-warmed LB medium without IPTG. C. Conjugation kinetics of pLS20cat after re-dilution of the donor cell culture. Conjugation kinetics using PKS7 and PKS11 as recipient and donor strains, respectively, was determined as described in Materials and Methods with the following modification. Overnight cultures were diluted, grown until late exponential growth phase (OD_600_ = 0,8), and diluted again (to OD_600_ = 0.05) before starting the experiment. B and C. t = 0 corresponds to the end of the exponential growth phase.

Many *rap* genes are transcriptionally coupled to a downstream-located *phr* gene. The small *phr* genes encode a product that, after being subjected to an export-import-maturation process, produces a mature penta- or hexapeptide that inhibits the activity of its cognate Rap protein. A putative *phr* gene, *phr*_LS20_, is located immediately downstream of *rap*_LS20_. The stop/start codons of these genes overlap and hence *phr_LS20_* is translationally coupled to *rap_LS20_*, a situation that is similar to those observed for some other *rap-phr* cassettes. Inspection of the deduced protein sequence suggests that *phr_LS20_* indeed encodes a typical pre-pro-peptide. The 44 residue gene product is predicted to contain an N-terminal signal peptide, a conserved motif upstream of its predicted maturation cleavage site, as well as conserved residues within the putative mature peptide [25], [43]. Based on this, the mature *phr*_LS20_–derived peptide is predicted to correspond to the five C-terminal residues of Phr*_LS20_, “QKGMY”, which we will refer to as Phr*_LS20_. To test a possible effect we determined conjugation efficiencies at the end of the exponential growth phase in the absence or presence of synthetic “QKGMY” peptide. The results presented in Figure 6A show that the presence of synthetic Phr*_LS20_ in the medium greatly reduced the maximum level of conjugation. These results support the view that Phr*_LS20_ inhibits Rap_LS20_–mediated de-repression of the conjugation genes. Conjugation efficiency did not alter significantly in the presence of another pentapeptide “EKAII”, demonstrating the specificity of the Phr*_LS20_ (not shown). The “EKAII” peptide is the predicted mature Phr*_576_ peptide encoded by a *rap-phr* cassette located on the related p576 plasmid [37].

Mature Phr* peptides encoded by other *rap-phr* cassettes are taken up by the oligo-peptide permease (Opp) of *B. subtilis* [30], [32], [42]. Figure 6A shows that Phr*_LS20_ forms no exception because the addition of Phr*_LS20_ peptide hardly affected conjugation when donor cells were *opp*-deficient.

The results of two further experiments provided additional evidence that Phr*_LS20_ inactivates Rap_LS20_. First, the Phr*_LS20_-mediated inhibition on conjugation was counteracted by ectopic expression of Rap_LS20_ (not shown). Second, we constructed a derivative of pLS20cat, pLS20phr, in which the *phr_LS20_* gene was deleted and tested its conjugation kinetics. The results presented in Figure 6B show that inactivation of *phr_LS20_* had similar effects on conjugation as those observed in the presence of ectopic expression of Rap_LS20_ (Figure 5) or inactivation of *rco_LS20_* (Figure 3). Thus, in the absence of *phr_LS20_* conjugation efficiencies are high and conjugation occurs during a very broad time window.

Under our laboratory conditions, efficient conjugation is limited to a rather small time window before the end of the exponential growth phase (see Figure 1). The results that Phr*_LS20_ inhibits the activity of Rap_LS20_, and that conjugation levels are high at all growth phases for pLS20phr indicate that the amount of Rap_LS20_ protein is not the limiting factor for activating conjugation but that its activity is inhibited by Phr*_LS20_ during early exponential as well as stationary growth phases. Phr*_LS20_-mediated inhibition of conjugation during stationary phase is most likely due to the accumulation of Phr*_LS20_ during growth, which will reach Rap_LS20_-inhibiting threshold levels at or near the end of the exponential growth phase. However, the low levels of conjugation during early exponential growth cannot be explained by a similar kind of reasoning because the freshly diluted culture will contain low levels of Phr*_LS20_ in the culture medium. One possible explanation for this is due to feasible intrinsic features of early exponential cells. This is very unlikely though taken into account that high levels of conjugation were obtained at early exponential growth phase with pLS20phr, pLS20rco or when Rap_LS20_ was ectopically expressed. An alternative explanation could be that Rap_LS20_-inhibiting levels of Phr*_LS20_ are still present inside the cells after overnight grown cultures are diluted in fresh medium. If this were the case, then high conjugation levels would be expected at early exponential growth phase by first growing the diluted overnight culture of donor cells to the end of the exponential growth phase and then diluting it again. The result of this experiment (Figure 6C) shows that high conjugation levels were indeed observed at early exponential growth phase under these conditions. Altogether, these results provide strong evidence that Phr*_LS20_ is the determining factor in regulating the time window at which conjugation genes are activated.

## Discussion

Here, we report for the first time the regulation of a conjugation system present on a native *B. subtilis* plasmid. Our results show that the conjugation genes of pLS20cat are not induced by recipient-produced pheromones, demonstrating that regulation of the conjugation system of pLS20cat is fundamentally different from that of the enterococcal plasmids pAD1 and pCF10.

Using different experimental approaches we demonstrated that the pLS20cat gene 27c encodes the master regulator of conjugation, Rco_LS20_. Interestingly, ectopic expression of Rco_LS20,_ predicted to be a DNA binding protein, resulted in the repression of not only the large, putative conjugation operon spanning genes 28 to 74, but also some other pLS20cat genes located outside the putative operon (for example, genes *11* and *16–21c*). While it is possible that the effects of Rco_LS20_ on the expression of some of the genes are indirect, the combination of our results clearly show that Rco_LS20_ is the master regulator of conjugation. Further work to characterize the DNA-binding properties of Rco_LS20_ and to identify the operator site(s) of Rco_LS20_ will be able to provide important information on how the different genes on pLS20 are regulated.

We also show that conjugation is activated by anti-repression and that Rap_LS20_, encoded by pLS20cat gene 25, is the anti-repressor of Rco_LS20_. Rap_LS20_ belongs to the large family of Rap proteins. At the moment of this writing, the number of *rap* genes present in databases exceeded 500 members. To our knowledge, this is the first time that a Rap protein has been demonstrated to activate plasmid conjugation.

Most *rap* genes are present on the genomes of bacilli. The genome of *B. subtilis* contains eleven *rap* genes. The majority of them inhibit directly or indirectly the activity of the transcriptional regulators that regulate processes such as sporulation, competence development and production of degradative enzymes and antibiotics [29]–[35]. *Rap* genes are also present on some rolling-circle and theta replicating *Bacillus* plasmids, and for those analyzed they too affect the production of extracellular proteases and sporulation [25], [36]–[40]. Surprisingly, our results showed that *rap_LS20_* plays no role in sporulation or competence.

**Figure 7:**
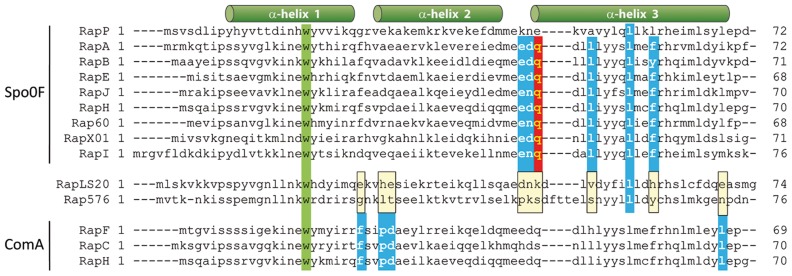
Conserved residues important for Rap proteins known to interact with Spo0F or ComA are not conserved in Rap_LS20_. Alignment of the N-terminal regions of *Bacillus* Rap proteins. In addition to Rap_LS20_ and Rap_576_, the alignment includes Rap proteins that previously have been demonstrated to dephosphorylate Spo0F (RapP, RapA, RapB, RapE, RapI, RapJ RapH, Rap_XO1_ ( = BXA0205), and Rap60 [Spo0F-phosphatase activity has not been demonstrated biochemically for Rap60]), and those shown to interact with ComA (RapF, RapC and RapH). Regions adapting an α-helical formation in RapH are indicated with green cylinders above the alignment. The highly conserved tryptophan residue present in all these Rap proteins is indicated in green. The catalytic Gln47 residue of RapH that is conserved in six of the seven other Spo0F-interacting Rap proteins as well as in RapI is highlighted in red. Alanine substitutions in Rap proteins that cause complete or significant loss of function/interaction with Spo0F and ComA are highlighted by blue boxes [44], [45]. RapH residue Leu55 is conserved in Rap_LS20_ and Rap_576_. It is worth mentioning that although the L55A mutant affected the function of RapH *in vivo*, no loss of RapH function was observed for this mutant *in vitro* [44]. Positions of the α-helices are indicated above the alignment.

Why Rap_LS20_ does not affect these differentiation routes may be explained by the recently obtained functional and structural data on how Rap proteins interact with regulatory proteins in the sporulation and competence pathways [44], [45]. Initiation of sporulation is controlled by the master regulator of sporulation, Spo0A, which becomes activated upon phosphorylation through phosphorelay. Eight of the Rap proteins encoded by the chromosome of *B. subtilis* and some Rap proteins encoded by *Bacillus* plasmids have been shown to interact with and dephosphorylate the sporulation protein Spo0F, one of the intermediate signal transducers. This interrupts the phosphate flux in which the phosphate is transferred from kinases to Spo0A through phosphorelay [46]. Competence development, on the other hand, is controlled by the transcription factor of competence, ComA. Previous studies have shown that RapC, RapF and RapH inhibit competence by interacting with ComA and preventing it from binding to DNA [35], [47], [48]. Probably all Rap proteins contain a rather small N-terminal domain of about 70 residues that is composed of a 3-helix bundle, a flexible linker, and a much larger C-terminal domain that generally harbors the Rap characteristic tetratricopeptide repeat (TPR) sequences [44], [45]. The recently resolved crystal structure of the Spo0F-RapH complex revealed that Spo0F interacts with both the C-terminal TPR domain and the N-terminal 3-helix bundle of RapH, including Gln47 in the N-terminal domain. This glutamine residue (GLu49 in the case of RapP encoded by the *B. subtilis* plasmid pBS32) is highly conserved and it constitutes the catalytic residue responsible for dephosphorylating Spo0F∼P [40], [44]. The alignment of the N-terminal regions of Rap proteins, presented in Figure 7, shows that neither the catalytic residue nor other residues in this region shown to be important for RapH phosphatase activity *in vitro* and *in vivo* are conserved in Rap_LS20_ or Rap_576_, the latter is encoded by a related theta replicating plasmid p576 [37]. Moreover, neither residues located in the C-terminal TPR domain shown to be important for RapH phosphatase activity are conserved in Rap_LS20_ and Rap_576_ (not shown).

In the case of ComA, several ComA-interacting residues of RapF, which are conserved among Rap proteins known to interact with ComA, have been identified and shown to be vital for the functionality of RapF [45]. The alignment in Figure 7 shows that these residues are not conserved in Rap_LS20_ or Rap_576_, consistent with our finding that Rap_LS20_ does not affect competence. Thus, residues important for interaction with Spo0F or ComA are not conserved in Rap_LS20_, which most probably explains why Rap_LS20_ does not affect sporulation or competence.

It is worth mentioning that Rap proteins involved in the regulation of the competence and sporulation pathways act as modulators, by inhibiting and/or delaying these developmental processes. On contrary, Rap_LS20_ functions as an activator, and rather than being a modulator, it plays a decisive role in the conjugation process by relieving Rco_LS20_-mediated repression. Thus, whereas conjugation levels were severely affected in the absence of *rap_LS20_*, conjugation was stimulated at all growth phases when Rap_LS20_ was ectopically expressed, accompanied by activation of the Rco_LS20_-repressed genes as analyzed by transcriptional profiling.

However, the ultimate determining factor responsible for defining the time window during which conjugation occurs is Phr*_LS20_. The observation that addition of synthetic Phr*_LS20_ peptide inhibits conjugation suggests that the peptide acts in cell-cell signaling rather than being an autocrine signal. Elevated conjugation levels were obtained at all growth phases for pLS20phr that lacked the *phr_LS20_* gene. These results strongly indicate that sufficient amounts of Rap_LS20_ are available to stimulate conjugation at all growth phases but that, under our standard laboratory conditions, its activity is inhibited by Phr*_LS20_ during early exponential and stationary growth phases, allowing efficient conjugation to occur only during a rather narrow time window near the end of the exponential growth phase.

The concentration of Phr*_LS20_ is expected to be high when all or the majority of the cells in a population produce the peptide, and low when the majority of the cells are not producing the peptide. In other words, conjugation genes will be activated specifically when donor cells are surrounded by recipient cells, and not by other donor cells. Besides preventing futile expression of conjugation genes when recipient cells are not present, strict regulation of the conjugation genes is likely to serve other purposes. For instance, pLS20cat replicates via the theta mode of replication [25]. During conjugation, however, replication switches to the rolling circle mode to generate the ssDNA strand that is destined to be transferred into the recipient cell. Simultaneous replication of the theta and rolling circle modes are likely not compatible and strict regulation of the conjugation genes, amongst which are those involved in initiating rolling circle replication, contributes to selecting the mode of replication according to the circumstances. In addition, it is likely that expression of the conjugation genes poses a large burden to the cell. This view is supported by our observations that growth is affected in cells harboring pLS20rco and pLS20phr, i.e. plasmids containing alterations leading to constitutive expression of the conjugation genes.

**Figure 8:**
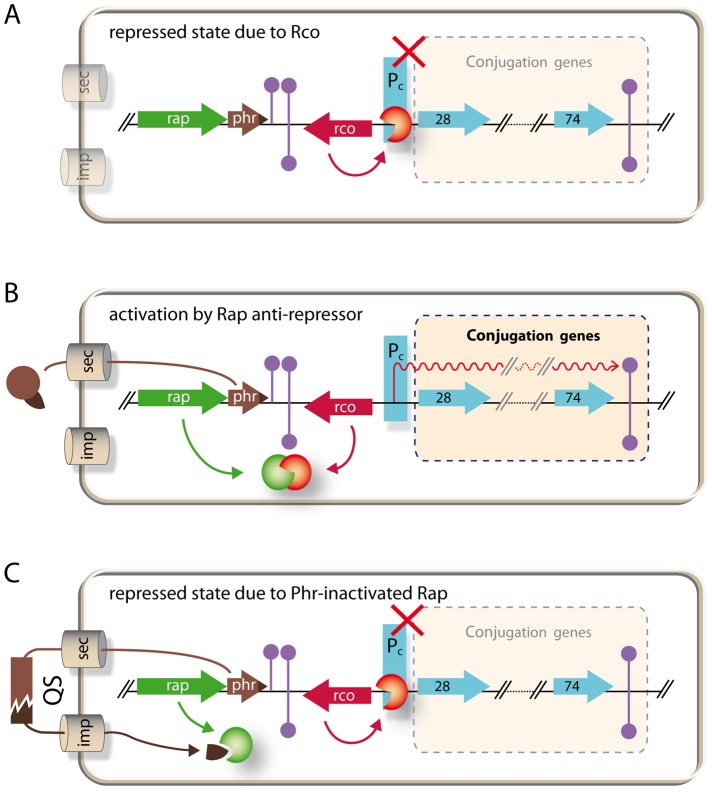
Model of regulatory circuitry of pLS20 conjugation genes. A. Repressed state due to Rco_LS20_. Gene *rco_LS20_* (red arrow, rco) encoding the master repressor of conjugation genes Rco_LS20_ is divergently transcribed from the putative conjugation operon encompassing genes 28 to 74 (light blue arrows). Rco_LS20_ inhibits expression of the conjugation genes by repressing a promoter, P_c_, located upstream of gene 28, the first gene of the putative conjugation operon (our unpublished results). B. Activation of conjugation by Rap_LS20_ anti-repressor. Gene _rapLS20_ (green arrow, rap) encodes the anti-repressor of Rco_LS20_ leading to de-repression of the conjugation genes. C. Repressed state due to inactivation of Rap_LS20_ by signaling peptide Phr*_LS20_. Gene *phr_LS20_* (brown arrow, phr) encodes a pre-pro-protein of 44 residues. This protein is subject to an export-maturation-import route. The mature pentapeptide inhibits activity of the Rap_LS20_ anti-repressor protein. For simplicity, import of the mature peptide has been shown into the cell producing the peptide. Grey cylinders labeled sec and imp, respectively, indicate the secretion and import routes. Extracellular processing of the secreted peptide is symbolized by the brown interrupted rectangle. QS, quorum sensing.

A summary of the regulatory circuitry of the pLS20 conjugation genes is schematically presented in Figure 8. Rco_LS20_ is responsible for maintaining conjugation in the default “OFF” state by repressing the conjugation genes. Rap_LS20_ can activate conjugation by relieving Rco_LS20_-mediated repression, but is only able to do so when its activity is not inhibited by the Phr*_LS20_ signaling peptide. Therefore, conjugation of the pLS20cat plasmid is strictly regulated by the Phr*_LS20_ peptide-mediated quorum sensing (QS) mechanism. QS is a common way by which bacteria communicate with one another using small and diffusible chemical signaling molecules. When the concentration of a signaling molecule reaches a certain “quorum”, bacteria respond by altering its gene expression profile at a (sub)population-wide scale (for review see, [49], [50]). Several cellular processes in both Gram+ positive and Gram- bacteria have been shown to be regulated by QS, among them the development of natural competence in *B. subtilis* and *Streptococcus pneumonia*, [30], [43], [50]. Here, we show that QS plays a role in HGT at another level by regulating expression of conjugation genes of plasmid pLS20. So far, QS has been reported to regulate conjugation genes of only a few other conjugative elements. These include the transfer of the tumor-inducing pTI plasmid of the Gram- *Agrobacterium tumefaciens* into plant cells. In this case, activation of conjugation requires two signaling peptides, one produced by the plant and the other by the donor cell [51]. Regulation of conjugation of the enterococcal plasmid pCF10, -and probably in a similar way pAD1-, also involves two signaling peptides, one produced by donor and the other by recipient cells. The two peptides compete for binding to a single transcriptional regulator, PrgX, and act antagonistically on conjugation. However, instead of being an activator, PrgX is a repressor. When PrgX is bound to the donor-produced signaling peptide the complex binds DNA and represses the conjugation genes. Conjugation genes become activated when recipient-produced signaling peptide replaces the donor-produced signaling peptide in the PrgX/peptide complex thereby inactivating the repressor activity of PrgX. Consequently, conjugation genes are activated by recipient produced signaling peptides [17]. Our results show that the QS mechanism to regulate conjugation genes of pLS20 differs in various aspects from those regulating conjugation of the pTi and pCF10/pAD1 plasmids. First, regulation of pLS20 conjugation genes involves not two but only one signaling peptide, Phr*_LS20_. Second, the signaling peptide does not act directly on the transcriptional regulator but instead regulates activity of another protein, Rap_LS20_, which functions as an anti-repressor. And third, the signaling peptide does not function to activate conjugation genes but to return the conjugation system to the default “OFF” state by inhibiting the activity of the anti-repressor.

Although *rap-phr* cassettes have not been shown before to regulate conjugation of a plasmid, the *B. subtilis* chromosomal *rapI-phrI* cassette has been described to regulate activation of the integrative conjugative element ICE*Bs1* [42]. There are several similarities but also interesting differences between the conjugation systems present on plasmid pLS20 and the chromosomal ICE*Bs1*. In both systems, transcription of the conjugation genes are repressed by an Xre-type repressor (Rco_LS20_ and ImmR, respectively) and the gene encoding the repressor protein is divergently oriented with respect to a large putative operon encoding the structural conjugation genes. In addition, in both cases conjugation is activated by a Rap protein (Rap_LS20_ and RapI, respectively) whose activity is controlled by a quorum sensing peptide encoded by the downstream *phr* gene. As we have proposed for *rap_LS20_-phr_LS20_*, a major function of the *rapI-phrI* cassette of ICE*Bs1* is a sensing mechanism to induce genes required for conjugation when recipient cells are present [42].

However, there are several important differences between the Rap_LS20_-Phr_LS20_ and the RapI-PhrI systems. One significant difference is that transfer of ICE*Bs1* requires, besides RapI, the ICE*Bs1*-encoded ImmA protein to relieve the ImmR-mediated repression of the ICE*Bs1* genes [52]. The *immA* gene is located immediately downstream of *immR*. ImmA is a protease that cleaves ImmR and its activity is probably controlled by RapI [53]. However, an *immA* homologue is not present on pLS20cat. More importantly, our preliminary results indicate that Rap_LS20_ functions directly as the anti-repressor of Rco_LS20_ (to be published elsewhere). Another major difference is that efficient mating of ICE*Bs1*, like most other ICEs, is limited to solid media, whereas pLS20 mates also efficiently in liquid medium [23], [24], [54, this study]. In a typical solid medium conjugation experiment, a concentrated mixture of donor and recipient cells is incubated on a solid surface for several hours, permitting donor cells to intimately contact recipient cells for a prolonged period of time. These conditions also correlate with high cell density, cell crowding and starvation, i.e. stationary phase conditions at which cells generally induce developmental pathways in order to cope with the suboptimal growth conditions.

The third difference is the timing of transfer. Auchtung *et al*. [42] reported that conjugation of ICE*Bs1* is low during mid exponential and much higher during stationary growth. The authors provided evidence that *rapI* is negatively regulated by the transition state regulator AbrB, which represses transcription of several *B. subtilis* genes specifically during the exponential growth phase [55]. Thus, RapI stimulates transfer of ICEBs1 during stationary phase conditions that favor intimate intercellular contacts; conditions that typically occur during conjugation on solid medium. In *B. subtilis* such conditions also stimulate initiation of the sporulation pathway. Most probably, sporulation and conjugative transfer of an ICE element are not compatible and hence efficient transfer of the ICE*Bs1* element would benefit from inhibiting sporulation in that cell. Interestingly, we have found that ectopic expression of RapI strongly affects sporulation. In agreement with our results, it has been shown very recently that RapI can dephosphorylate Spo0F *in vitro* [56]. Together these results demonstrated that RapI has a dual function: it activates transfer of ICE*Bs1* during stationary phase and inhibits the initiation of sporulation that is normally stimulated under these conditions.

We have shown that Rap_LS20_ regulates conjugation of pLS20cat in a strikingly different manner. Several results showed that efficient pLS20cat conjugation occurs during exponential growth and that it is strongly inhibited during stationary growth. This important difference may be related to the fact that conjugation of pLS20cat occurs efficiently in liquid medium when cells have a planktonic lifestyle and probably spend more time in the exponential growth phase than cells growing in sessile communities. Our results on Rap_LS20_, together with published results on other Rap proteins, demonstrate the enormous plasticity of how these proteins have evolved into versatile regulatory proteins that control diverse cellular processes by interacting with a wide range of other regulatory proteins.

## Materials and Methods

### Bacterial strains, media, oligonucleotides and peptides

*Escherichia coli* and *B. subtilis* strains were grown in Luria-Bertani (LB) medium or on 1.5% LB agar plates [57]. When appropriate, media or agar plates were supplemented with the following antibiotics: ampicillin (100 µg/ml), erythromycin (1 and 150 µg/ml for *B. subtilis* and *E. coli*, respectively), chloramphenicol (5 µg/ml), spectinomycin (100 µg/ml), kanamycin (10 µg/ml). Competent cells were prepared as described before [58]. Transformants were selected on LB agar plates with appropriate antibiotics. For sporulation experiments, *Bacillus* strains were grown in Schaeffer's medium [59]. Plasmids and strains used are listed in supplemental Table S4. *B. subtilis* strains are all isogenic with *B. subtilis* strain 168 (Bacillus Genetic Stock Centre Code 1A700). Oligonucleotides used (Isogen Life Sciences, The Netherlands) are listed in supplemental Table S5. Phr*_LS20_ and Phr*_576_ peptides were synthesized by the Proteomics department of our Institute.

### Transformation

*E. coli* cells were transformed using standardized methods [57]. For standard *B. subtilis* transformations, competent cells were prepared as described by Bron (1990). For making knockout version of pLS20cat, high competency protocol was used as described by Zhang and Zhang [60].

### Construction of plasmids and strains

DNA techniques were performed using standard molecular methods [57]. All enzymes used were purchased from New England Biolabs, USA. The correctness of all constructs was verified by sequence analysis. To construct a strain containing *rco*_LS20_ gene under the control of the IPTG-inducible P_spank_ promoter, the gene was amplified from plasmid pLS20cat by polymerase chain reaction (PCR), using primers Xre20UpHind and Xre20DnNhe. The PCR product was cleaved with *Hin*dIII and *Nhe*I and cloned into these sites of vector pDR110 (a gift from D. Rudner, see Table S4) to produce pDRrco_LS20_. Plasmid pDR110 is a *B. subtilis amyE* integration vector that contains a multiple cloning site located behind the IPTG-inducible P*_spank_* promoter. Next, the P_spank-_ *rco_LS20_* construct was placed at *amyE* locus at the *B. subtilis* chromosome by transforming competent *B. subtilis* 168 cells with plasmid DNA pDRrco_LS20_ and selecting for spectinomycin resistant colonies. Double cross over event of the resulting strain PKS9 was confirmed by the loss of a functional amylase gene. Plasmid pLS20cat was conjugated into strain PKS9 to give strain PKS14. The same strategy, using primers Rap20UpSal and Rap20DnNhe, was applied to obtain strain GR20 that contains a P_spank-_ *rap_LS20_* fusion at the *amyE* locus. GR23 strain was obtained by conjugating plasmid pLS20cat into strain GR20. In plasmid pPKS26 *rapI* is placed under the control of the P_hyspank_ promoter. This plasmid was constructed by first amplifying a *rapI* containing DNA fragment by PCR using oligos oGR85 and oGR86 and *B. subtilis* 168 DNA as template. Next the PCR fragment was digested with *Nhe*I and *Sph*I and cloned in vector pDR111 digested with the same enzymes. Finally, the P_hyspank-_ *rapI* construct was placed at *amyE* locus of the ICEBs1 negative strain PY79 by using plasmid pPKS26 to transform competent PY79 cells resulting in strain PKS139. A standard protocol was used to construct derivatives of pLS20cat in which the *rap_LS20_*, *phr_LS20_ or rco_LS20_* genes were replaced by an antibiotic resistance marker [37].

### Conjugation assays

Unless specified otherwise, conjugation was carried out in liquid medium as described by Itaya et al. [24]. Thus, for standard conjugation experiments, overnight cultures of donor and recipient cells, grown in the presence of appropriate antibiotics, were diluted 25 fold in fresh 37°C pre-warmed LB medium without antibiotics and grown for 2.5 h in shaking (125 rpm) water bath. Next, 200 µl of both donor and recipient cells were mixed in 2.5 ml eppendorf tube and incubated for 15 min at 37°C without shaking to permit conjugation. Finally, appropriate dilutions were plated on LB agar plates supplemented with proper antibiotics to select either for transconjugants or for donor cells. When conjugation efficiencies were determined as a function of growth, overnight cultures were diluted to an OD_600_ of 0.01. Next, donor and recipient cells were grown separately (180 rpm) and 200 µl of the donor and recipient cultures were withdrawn at different times and proceeded as described above. Growth was followed by measuring OD_600_ at regular intervals. In order to study the effect on conjugation of over-expression of a given gene placed under the control of the inducible P_spank_ promoter, IPTG was added to prewarmed LB medium used for inoculation of the overnight grown cultures. Unless mentioned otherwise, IPTG was added to a final concentration of 1 mM.

All conjugation experiments were repeated at least three times. The entry into stationary growth (t = 0) is determined in retrospect based on the growth curve. Consequently, time points at which samples were taken fluctuate slightly between each experiment. Values for specific time points extrapolated from the curves of repeated experiments showed that they differed by less than 10%. Therefore, the results of representative experiments are presented in Figures 1, 3, 5 and 6.

### RNA isolation and RNA sequencing

Total RNA was isolated from late exponentially growing cells by using RNeasy Mini Kit from Qiagen according to manufacturer's protocol. RNA protect solution provided by Qiagen was used to ensure the integrity of RNA during isolation and also to stop transcription at given time points. RNA was treated with DNAseTurbo (Ambion) to remove possible traces of contaminant DNA. Between 5 to 15 µg of total RNA was subjected to rRNA removal using RiboZero (Epicentre, either Gram-positive specific or metabacteria-specific) following the manufacturer instructions to obtain 150–250 ng of rRNA-depleted RNA. Next, RNA of each sample was used to prepare cDNA libraries using a procedure that preserves information about transcript's direction (ScriptSeq mRNA library preparation kit, Illumina compatible; Epicentre) [61]. As specified by the supplier, samples were fragmented for 5 min at 85°C and subsequently bar-coded so that they could be run in combination.

After library prep, samples were titrated by quantitative PCR, pooled and bound at a final concentration of about 10 pM to an Illumina SR-flowcell using a Cluster Station apparatus (Illumina). Libraries were then run on a GAiix equipment (Unidad de Genómica, Parque Científico de Madrid) by SBS under a single-read 1×36 protocol. Quality filtering was performed automatically according to Illumina specifications and fastq files generated.

### Bioinformatic analysis of RNAseq data

#### Data set

The analyzed data set was constituted by five *B. subtilis* subsp. *subtilis* str. 168 and plasmid pLS20cat samples corresponding to four different experimental conditions (see supplemental Table S6), with a total of 56,439,165 single end reads of 36 nt length in FASTQ format. Data were analyzed using the standard bioinformatic analysis workflow of a RNA-seq experiment detailed below.

#### Reads quality:

A preliminary analysis of the quality of the reads was performed using FastQC, a Java tool with graphic interface (http://www.bioinformatics.babraham.ac.uk/projects/fastqc/). Percentages between 93.02% and 93.24% of all bases had a quality score of 30 or higher (probability of incorrect base call of 10^−3^ or lower) and between 85.43% and 85.85% of all bases had a quality score of 35 or higher (probability of incorrect base call of 3·10^−4^ or lower), being 40 the maximum score quality reported in FASTQ format (probability of incorrect base call of 10^−4^ or lower). Because of the high quality it was not necessary to process the reads by filtering or trimming them. The results are summarized in supplemental Table S6.

#### Alignment:

 The reads were mapped to the published *B. subtilis* subsp. *subtilis* str. 168 and plasmid pLS20cat reference genomes using Bowtie software [62] with the following parameters. Maximum allowed number of mismatches 3, input qualities are Phred+33 [63], [64], and the “-–best” option was switched on, ensuring that reported alignments are “best” in terms of chosen criteria (allowed number of mismatches), and that alignments are reported in best-to-worst order. Of the total reads, a percentage between 92.48 and 98.51% could be mapped to the reference genome with 79 to 106-fold sequencing coverage across the entire genome. Unmapped reads were searched in UniVec database using BLAST [65]. UniVec is a database that contains DNA sequences of cloning/expression vectors, adapters, linkers, and primers that are commonly used in the process of cloning and sequencing nucleic acids (http://www.ncbi.nlm.nih.gov/tools/vecscreen/univec/). This database was used to identify such contaminating sequences from the unmapped reads. Of total reads, percentages between 0.18% and 0.56% were assigned to UniVec database sequences, revealing very low levels of vector contamination. Unmapped reads were discarded for further analysis. These results were summarized in supplemental Table S6. Out of the total of 56,439,165 reads, 1,596,385 (2.83%) mapped to the pLS20cat genome, which were used to calculate expression levels of individual pLS20cat genes under the different conditions.

#### Expression levels:

The alignment files were processed using EpiCenter software (http://www.niehs.nih.gov/research/resources/software/biostatistics/epicenter/), an analysis tool of genome-wide mRNA-seq or ChIP-seq data for detecting differentially expressed genes [66].

Plasmid pLS20cat expression levels were additionally used to draw a heat map, by using Matrix2png software (http://www.chibi.ubc.ca/matrix2png/) [67], that graphically shows the expression levels of “wild type” experimental conditions (left lane on Figure 4). In addition, the heatmap shows the differences in expression of pLS20cat genes when Rco_LS20_ or Rap_LS20_ were ectopically expressed (middle and right lanes Figure 4, respectively) compared to the wild type situation.

### Computer-assisted analysis

Protein blast (blastP and psi-blast) searches (http://blast.ncbi.nlm.nih.gov/Blast.cgi) were performed for each ORF of pLS20cat to gain insights in the function of the proteins encoded by these ORFs. Alignments of the primary amino acid sequences of Rco homologues were made using the ClustalW2 program (http://www.clustal.org/clustal2). Adobe Photoshop CS2 and Adobe Illustrator were used for creating figures and art work. The Excel program was used to create graphics.

## Supporting Information

Figure S1 Alignment of different Xre-type repressors. Helix-Turn-Helix region is highlighted in red. Conserved residues (present in at least 6 of the 10 proteins) are highlighted in yellow. Abbreviations (accession numbers given in brackets): DBHTH_Paeni.HGF7, DNA-binding helix-turn-helix protein of *Paenibacillus sp.* HGF7 (ZP_08510432); Xre_Paeni.polySC2, XRE family transcriptional regulator *Paenibacillus polymyxa* SC2 (YP_003945377); Rco_LS20,_ Repressor of conjugation B. *subtilis* natto IFO 3335 plasmid pLS20 (YP_004243490); Xre_p576, Xre type repressor *B. pumilus* NRS576 plasmid p576; DBP_B.subp19, DNA binding protein of plasmid p19 of *B. subtilis* 19 (ABP52080); Regulator_P.elgii, transcriptional regulator *Paenibacillus elgii* B69 (ZP_09077606); Repressor_B.amylo, transcriptional repressor RghR of *B. amyloliquefaciens* DSM7 (YP_003921816); Xre_Dehalo.GT, XRE family transcriptional regulator *Dehalococcoides* sp. GT (YP_00346200); Regulator_Desulfo.DSM, putative transcriptional regulator *Desulfosporosinus youngiae* DSM 17734(ZP_09652311); ImmR_ICE168, XRE family transcriptional regulator of ICE element *B .subtilis* 168 (NP_388363).
(DOCX)

Table S1 Characteristics of genes and ORFs located in the putative conjugation operon.
(DOCX)

Table S2 Ectopic expression of Rap_LS20_ does not affect competence or sporulation.
(DOCX)

Table S3 ICE*Bs1* encoded RapI inhibits sporulation.
(DOCX)

Table S4 Strains and plasmids used in these studies.
(DOCX)

Table S5 Oligonucleotides used in these studies.
(DOCX)

Table S6 Summary of experimental RNA seq conditions, sequence reads, coverage, and quality score.
(DOCX)
