# Supplementary figures and images for: Mobility of the Native Bacillus subtilis Conjugative Plasmid pLS20 Is Regulated by Intercellular Signaling
Source: PLoS Genet. 2013 Oct 31;9(10):e1003892. doi: 10.1371/journal.pgen.1003892 (PMC3814332; doi:10.1371/journal.pgen.1003892)

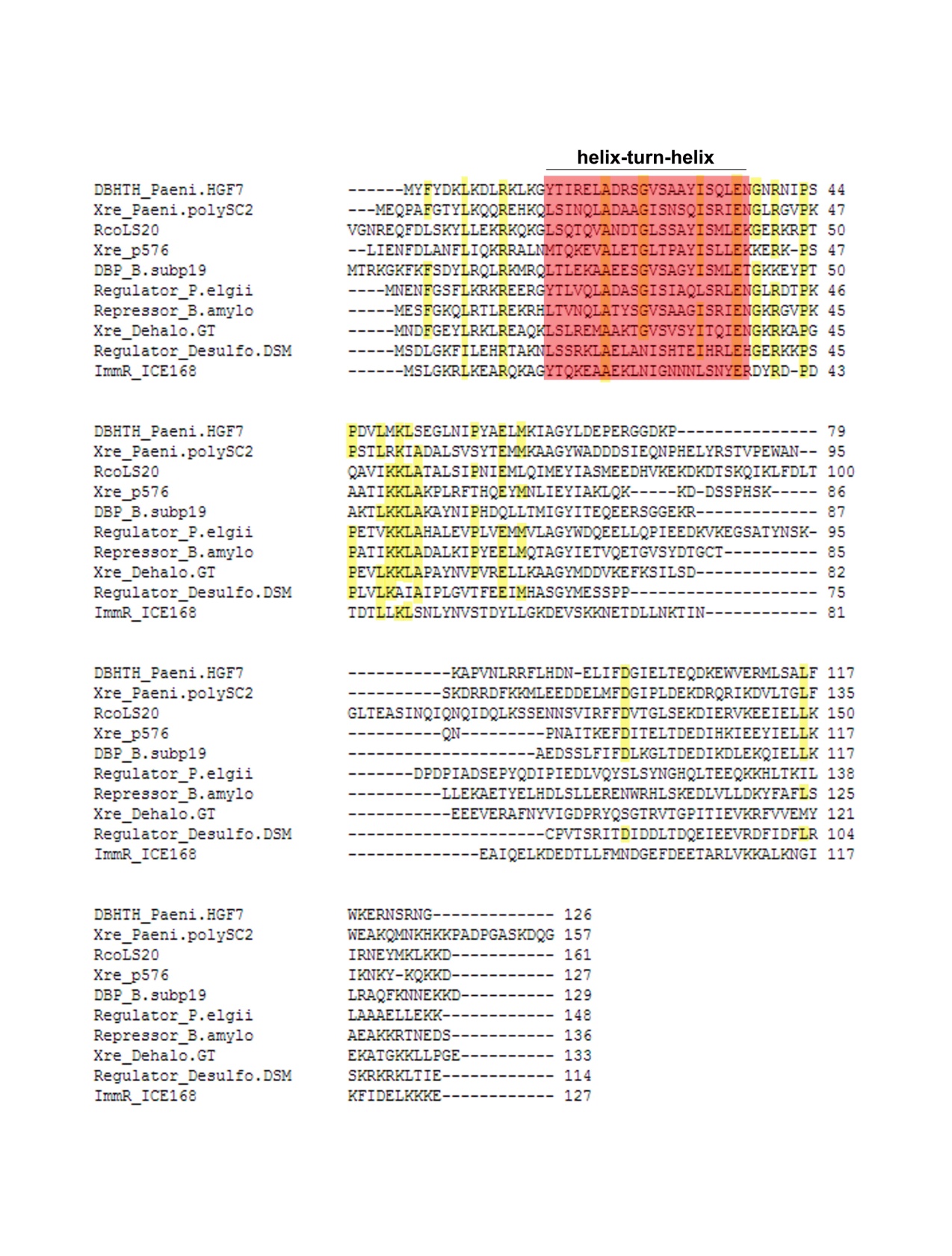

Supplement: Figure S1 — Alignment of different Xre-type repressors. Helix-Turn-Helix region is highlighted in red. Conserved residues (present in at least 6 of the 10 proteins) are highlighted in yellow. Abbreviations (accession numbers given in brackets): DBHTH_Paeni.HGF7, DNA-binding helix-turn-helix protein of Paenibacillus sp. HGF7 (ZP_08510432); Xre_Paeni.polySC2, XRE family transcriptional regulator Paenibacillus polymyxa SC2 (YP_003945377); RcoLS20, Repressor of conjugation B. subtilis natto IFO 3335 plasmid pLS20 (YP_004243490); Xre_p576, Xre type repressor B. pumilus NRS576 plasmid p576; DBP_B.subp19, DNA binding protein of plasmid p19 of B. subtilis 19 (ABP52080); Regulator_P.elgii, transcriptional regulator Paenibacillus elgii B69 (ZP_09077606); Repressor_B.amylo, transcriptional repressor RghR of B. amyloliquefaciens DSM7 (YP_003921816); Xre_Dehalo.GT, XRE family transcriptional regulator Dehalococcoides sp. GT (YP_00346200); Regulator_Desulfo.DSM, putative transcriptional regulator Desulfosporosinus youngiae DSM 17734(ZP_09652311); ImmR_ICE168, XRE family transcriptional regulator of ICE element B .subtilis 168 (NP_388363). (DOCX) [file pgen.1003892.s001.docx]
