# Supplementary material for: Mobility of the Native Bacillus subtilis Conjugative Plasmid pLS20 Is Regulated by Intercellular Signaling
Source: PLoS Genet. 2013 Oct 31;9(10):e1003892. doi: 10.1371/journal.pgen.1003892 (PMC3814332; doi:10.1371/journal.pgen.1003892)
Supplement: Table S1 — Characteristics of genes and ORFs located in the putative conjugation operon. (DOCX) [file pgen.1003892.s002.docx]

| **Table S1**. Characteristics of genes and ORFs located in the putative conjugation operon | | | | |
| --- | --- | --- | --- | --- |
| **ORF** | **Start-stop** | **Size (aa)** | **Putative function and/or most relevant homology** | **Putative RBS** |
|  |  |  | **-Microorganism (amino acid identity %)** |  |
|  |  |  | **-Putative conserved domain (PCD)** |  |
| 25 | 75-1181 | 368 | Rap (response regulator aspartate phosphatase)  TPR superfamily domain | GGAGG-7-ATG |
| 26 | 1178-1312 | 44 | Putative prepro-Phr signaling peptide | AACGGAGG-7-ATG |
| 27c | 1483-1968 | 161 | Repressor protein  HTH-XRE superfamily | GAGGTGG-5-GTG |
| 28 | 2564-3082 | 172 | Hypothetical protein LGRDSM20601_p0002  *Listeria grayi* (24%) | AGGGAGG-7-TTG |
| 29 | 3466-4638 | 390 | Hypothetical protein LMOh7858_pLM80_0004  *Listeria monocytogenes* str. 4b H7858 (29%) | AAAGGTGT-11-ATG |
| 30 | 4705-4941 | 99 | Pirin domain protein  *Pseudomonas fulva* 12-X (32%)  Tfp pilus assembly protein PilV | GCTGG-7-GTG |
| 31 | 4938-5273 | 111 | Hypothetical protein HMPREF1012_02441  *Bacillus* sp. BT1B_CT2 (70%)  Prokaryotic membrane lipoprotein lipid attachment site | AAGAACG-6-TTG |
| 32 | 5334-5822 | 162 | Hypothetical protein BCAH1134_A0022  *Bacillus cereus* AH1134 (37%) | AAAGGAGA-5-ATG |
| 33 | 6124-6336 | 70 | ABC transporter permease  *Serratia plymuthica* PRI-2C (30%) | AAATGAGGTGG-4-ATG |
| 34 | 6519-8780 | 778 | Conserved hypothetical protein  *L. monocytogenes* str. 4b H7858 (53%) | GCAGCGC-8-GTG |
| 34A | 8808-9008 | 98 | Pectate lyase  *Bacteroides finegoldii* DSM 17565 (30%) | TTTGGAGG-7-ATG |
| 35 | 9036-9398 | 120 | Sensor histidine kinase GraS  *Staphylococcus epidermidis* NIHLM020 (23%) | AAAGGGAAGG-2-ATG |
| 36 | 9446-9826 | 126 | Hypothetical protein LM5578_p34  *L. monocytogenes* 08-5578 (46%) | AAATCGAGG-7-ATG |
| 37 | 9839-11047 | 402 | Type II/IV secretion system protein  Listeria monocytogenes str. 4b H7858 (51%)  Flp pilus assembly protein, ATPase CpaF multidomain | AGGAGG-7-ATG |
| 38 | 11049-11852 | 267 | Hypothetical protein bcere0018_53120  *B. cereus* Rock1-15 (46%) | AGGGGG-5-GTG |
| 39 | 11849-12586 | 245 | conserved hypothetical protein  *L. monocytogenes* str. 4b H7858 (42%) | AATGGAGG-7-ATG |
| 40 | 12609-12971 | 120 | Conserved hypothetical protein  *L. grayi* DSM 20601 (44%)  Domain of unknown function (DUF4320) | AAAGAGG-8-ATG |
| 40A | 13049-13162 | 37 | Hypothetical protein NT03LS_3239  *Listeria seeligeri* FSL N1-067 (45%) | AGGATG-6-GTG |
| 41 | 13191-13334 | 47 | Hypothetical protein pAW63_024  *Bacillus thuringiensis* serovar kurstaki (53%) | AGGAGA-8-ATG |
| 42 | 13361-13504 | 47 | Hypothetical protein pAW63_024  *B. thuringiensis* serovar kurstaki (53%) | AGGAGA-9-ATG |
| 43 | 13560-13775 | 71 | Hypothetical protein | AGGCGG-7-ATG |
| 44 | 13806-13997 | 63 | Hypothetical protein | AAGGAGG-8-ATG |
| 45 | 14035-14265 | 76 | Hypothetical protein TEH_20450  *Tetragenococcus halophilus* NBRC 12172 (43%) | AAGAGG-8-ATG |
| 46 | 14286-15743 | 485 | Hypothetical protein | GGGGG-7-ATG |
| 47 | 15770-16861 | 363 | Hypothetical protein | AAAGGAGG-5-TTG |
| 48 | 16910-19270 | 786 | VirD4 component of type IV secretory pathway | TGAGG-7-TTG |
| 49 | 19389-22208 | 974 | Transmembrane protein  *L. monocytogenes* FSL J1-208 (42%) | GGTGG-6-ATG |
| 50 | 22205-22516 | 103 | Hypothetical protein LmonocyFSL_00185  *L. monocytogenes* FSL J1-208 (34%) | AAAGGGGG-9-ATG |
| 51 | 22518-23096 | 192 | TraE, Type IV secretion system protein  *L. monocytogenes* FSL J1-208 (33%) | AGGAGG-6-ATG |
| 52 | 23110-25005 | 631 | TraE, Type IV secretion system protein  *L. monocytogenes* FSL J1-208 (47%) | AGGCGG-9-ATG |
| 53 | 25005-25631 | 208 | Putative lipoprotein LpqB  *L. monocytogenes* FSL J1-208 (24%) | AAGGTGG-6-ATG |
| 54 | 25624-26730 | 368 | Putative lipoprotein  *L. monocytogenes* FSL J1-208 (56%)  Lysozyme_Like superfamily and NLPC_P60 superfamily | AAAGGGTTG-7-ATG |
| 55 | 26749-27558 | 269 | Hypothetical protein HA1_05612  *Clostridium perfringens* F262 (32%) | AAAGGAGG-12-GTG |
| 56 | 27893-28132 | 79 | CopG-like DNA binding protein  Hypothetical protein LMIV_p039  *L. monocytogenes* FSL J1-208 (32%) | AAAGGAGC-8-ATG |
| 57 | 28315-28758 | 147 | Hypothetical protein LmonocyFSL_00140  *L. monocytogenes* FSL J1-208 (37%) | TGAGG-9-ATG |
| 58 | 28758-29990 | 410 | Hypothetical protein LmonocyFSL_00135  Putative Relaxase  *L. monocytogenes* FSL J1-208 (52%) | AAAGGTGA-6-ATG |
| 59 | 30021-30491 | 156 | Hypothetical protein CJD_A0352  *C. perfringens* D str. JGS1721 (29%) | AAAGGAGA-8-ATG |
| 60 | 30566-31945 | 459 | Zinc beta-ribbon domain containing protein  *L. monocytogenes* FSL J1-208 (42%)  Superfamily :Topoisomerase-primase domain,  DNA primase, catalytic core | AAAGGGGG-7-ATG |
| 61 | 32052-32207 | 51 | Hypothetical protein  *Bacillus amyloliquefaciens* LL3 (63%) | AAAGGGAGG-8-ATG |
| 62 | 32208-32720 | 170 | Toxin-antitoxin system, toxin component, MazF family *Streptococcus mitis* bv. 2 str. SK95 (37%) | AGGAAT-8-TTG |
| J43 | 32727-32879 | 50 | Hypothetical protein | AACGGTAA-6-TTG |
| 63 | 32938-33285 | 115 | Hypothetical protein Bsb_33  *B. subtilis* (28%) | AAGGGAG-8-TTG |
| 63Ac | 33547-33657 | 36 | Serine/threonine-protein kinase  *Lentisphaera araneosa* HTCC2155 (42%) | AAAGGGGG-7-ATG |
| 64 | 33969-34367 | 132 | Rok, ComK repressor  *B. subtilis* 168 | AAAGGAGA-7-ATG |
| 65 | 34536-34829 | 97 | Transcriptional regulator  *Desulfomonile tiedjei* DSM 6799 (29%) | AAAAGGGGT-6-ATG |
| 66 | 34823-35293 | 156 | Single-strand DNA binding protein  *B. subtilis* subsp. natto  Ssb superfamily protein | GGAGGG-9-ATG |
| 67 | 35318-35767 | 149 | Hypothetical protein bthur0013_63530  *B. thuringiensis* IBL 200 (28%) | AAAGGAGA-9-ATG |
| 68 | 35882-36097 | 71 | Putative serine/threonine sodium symporter  *B. subtilis* subsp. natto | AGGAGG-7-ATG |
| 69 | 36219-36788 | 189 | Recombinase/integrase  *Enterococcus casseliflavus* EC30(49%) | AAGAGG-8-ATG |
| 70 | 36817-37056 | 79 | ABC-type Mn2+/Zn2+ transport systems permease component *Bifidobacterium animalis* sub (36%) | AGGGGG-5-ATG |
| 71 | 37069-37731 | 220 | Hypothetical protein | AAAGGAGA-4-ATG |
| 72 | 37794-38111 | 105 | YhgE/Pip C-terminal domain protein  *Eggerthella* sp. HGA1 (23%) | AAGGGG-11-ATG |
|  | 38114-38173 | 19 | Hypothetical protein | AAGAGG-7-TTG |
| 73 | 38388-39431 | 347 | Hypothetical protein  *Paenibacillus* sp. JDR-2 (23%) | GGAAG-9-ATG |
| 74 | 39424-39669 | 81 | rplI gene product  *Candidatus Blochmannia pennsylvanicus* str. BPEN (31%) | AGGAGG-8-ATG |
