# Supplementary material for: Mobility of the Native Bacillus subtilis Conjugative Plasmid pLS20 Is Regulated by Intercellular Signaling
Source: PLoS Genet. 2013 Oct 31;9(10):e1003892. doi: 10.1371/journal.pgen.1003892 (PMC3814332; doi:10.1371/journal.pgen.1003892)
Supplement: Table S2 — Ectopic expression of RapLS20 does not affect competence or sporulation. (DOCX) [file pgen.1003892.s003.docx]

| **Table S2.** Ectopic expression of Rap_LS20_ does not affect competence or sporulation | | |
| --- | --- | --- |
| Process | IPTG (1mM) | Relative efficiency |
| Competence | - | 1 |
|  | + | 2.638 |
| sporulation | - | 1 |
|  | + | 0.65 |
| Strain GR20 (*amyE*::P_spank_-*rap_LS20_*) was used to determine efficiencies of competence and sporulation using standard protocols. Experiments were carried out twice and the differences between experiments were less than 10% | | |
