# Supplementary material for: Mobility of the Native Bacillus subtilis Conjugative Plasmid pLS20 Is Regulated by Intercellular Signaling
Source: PLoS Genet. 2013 Oct 31;9(10):e1003892. doi: 10.1371/journal.pgen.1003892 (PMC3814332; doi:10.1371/journal.pgen.1003892)
Supplement: Table S3 — ICEBs1 encoded RapI inhibits sporulation. (DOCX) [file pgen.1003892.s004.docx]

| **Table S3.** ICE*Bs1* encoded RapI inhibits sporulation | | |
| --- | --- | --- |
| Strain | IPTG (1mM) | Relative sporulation efficiency |
| wt | - | 1. |
|  | + | 1.268 |
| PKS139 | - | 0.85 |
|  | + | 0.004 |
| Strain PKS139 (*amyE*::P_hyspank_-*rapI*) was used to determine sporulation efficiencies. Experiments were carried out twice and the differences between experiments were less than 10% | | |
