# Supplementary material for: Mobility of the Native Bacillus subtilis Conjugative Plasmid pLS20 Is Regulated by Intercellular Signaling
Source: PLoS Genet. 2013 Oct 31;9(10):e1003892. doi: 10.1371/journal.pgen.1003892 (PMC3814332; doi:10.1371/journal.pgen.1003892)
Supplement: Table S4 — Strains and plasmids used in these studies. (DOCX) [file pgen.1003892.s005.docx]

| **Table S4.** Strains and plasmids used | | | |
| --- | --- | --- | --- |
| ***Strains*** | | **Genotype or description** | **Reference or source** |
| *E. coli* | |  |  |
|  | XL1-Blue | *end*A1 *gyr*A96(nal^R^) *thi*-1 *rec*A1 *rel*A1 *lac* *gln*V44 F'[ ::Tn10 *pro*AB^+^ *lac*I^q^ Δ(*lac*Z)M15] *hsd*R17(r_K_^-^ m_K_^+^) | [68] |
| *B. subtilis* | |  |  |
|  | BEST40401 | *hsdR hsdM leu arg* containing plasmid pLS20cat | [24] |
|  | 1A976 | *his npr*E18 *apr*E3 *egl*S Δ102 *bgl*T/*bgl*S ΔEV *lac*A::P_xylA_-*comK* (em) | [60], BGSC* |
|  | AK3 | *oppA*::Tn10 (spc) | [69] |
|  | 168 (1A700) | *trpC2* | BGSC |
|  | PY79 (1A747) | prototrophic attICEBs1 attSPbeta | BGSC |
|  | PKS11 | *trpC2* containing plasmid pLS20cat | [27] |
|  | PKS56 | *his* *npr*E18 *apr*E3 *egl*S Δ102 *bgl*T/*bgl*S ΔEV *lac*A::P_xylA_-*comK* (em) containing plasmid pLS20cat | [27] |
|  | PKS7 | *trpC2* *thrC*::Δ *Lac*Z(em) | This work |
|  | PKS9 | *trpC2 amy*E::P_spank_*rco*_LS20_ (Spc) | This work |
|  | PKS14 | *trpC2*  *amy*E::P_spank_*rco*_LS20_ (Spc) containing plasmid pLS20cat | This work |
|  | GR20 | *trpC2* *amy*E::P*_spank_* *rap_LS20_* (spec) | This work |
|  | GR23 | *trpC2* *amy*E::P*_spank_* *rap_LS20_* (spec) containing plasmid pLS20cat | This work |
|  | PKS57 | *his* *npr*E18 *apr*E3 *egl*S Δ102 *bgl*T/*bgl*S ΔEV *lac*A::P_xylA_-*comK* (em)  *amyE::P_spank_rco_LS20_(spec)* | This work |
|  | PKS58 | *his npr*E18 *apr*E3 *egl*S Δ102 *bgl*T/*bgl*S ΔEV *lac*A::P_xylA_-*comK* (em)  *amyE::P_spank_rco_LS20_(spec)*  containing pLS20cat plasmid | This work |
|  | PKS59 | *his npr*E18 *apr*E3 *egl*S Δ102 *bgl*T/*bgl*S ΔEV *lac*A::P_xylA_-*comK* (em) *amyE::P_spank_rco_LS20_(spec)* containing pLS20rco plasmid | This work |
|  | PKS77 | *his npr*E18 *apr*E3 *egl*S Δ102 *bgl*T/*bgl*S ΔEV *lac*A::P_xylA_-*comK* (em) contaning plasmid pLS20rap | This work |
|  | PKS79 | *trpC2* containing plasmid pLS20rap | This work |
|  | PKS87 | *trpC2*  *amy*E*::P_spank_ rap_LS20_(spec)* containing plasmid pLS20rap | This work |
|  | PKS86 | *trpC2*  *amy*E::P_spank_rco_LS20_ (spec) containing pLS20rco | This work |
|  | PKS97 | *trpC2*  *oppA::Tn10 (Spec)* | This work |
|  | PKS98 | *trpC2*  *oppA::Tn10 (Spec)*containing plasmid pLS20cat | This work |
|  | PS110 | *trpC2 amyE::*P_spank_*-Δ(spec)* | This work |
|  | PKS113 | *his npr*E18 *apr*E3 *egl*S Δ102 *bgl*T/*bgl*S ΔEV *lac*A::P_xylA_-*comK* (Em) *amy*E::*P_spank_rco*_LS20_(*spec*) containing plasmid pLS20phr | This work |
|  | PKS117 | *trpC2* *amy*E::*P_spank_rco*_LS20_(*spec*) containing plasmid pLS20phr | This work |
|  | PKS139 | Prototrophic, attICEBs1 attSPbeta amyE::*P_hspank_rapI* (*spec*) | This work |
| **Plasmids** | | **Description** | **Reference or source** |
|  | pDR110 | *B. subtilis amyE* integration vector containing IPTG-inducible P*_spank_* promoter | D. Rudner |
|  | pBEST501 | *E. coli* vector containing neomycin resistance marker in multiple cloning site | [70] |
|  | pDG1663 | B. subtilis *thrC* integration vector containing promoter-less *lacZ* gene which is used for promoter screening | [71], BGSC |
|  | pLS20cat | Native plasmid pLS20 labelled with Cm resistance cassette in unique *Sal*I site. | [24] |
|  | pDRrco_LS20_ | *rco_LS20_* gene cloned in integration vector pDR110 | This work |
|  | pDRrap_LS20_ | *rap_LS20_* gene cloned in integration vector pDR110 | This work |
|  | pLS20xre | pLS20cat derivative in which *xre_LS20_* gene is replaced by Neomycin marker | This work |
|  | pLS20rap | pLS20cat derivative in which *rap_LS20_* gene is replaced by Neomycin marker | This work |
|  | pLS20phr | pLS20cat derivative in which *phr_LS20_* gene is replaced by Neomycin marker | This work |
|  | pPKS26 | *rapI* gene is cloned in the integration vector pDR111 | This work |
| *, BGSC: Bacillus Genetic Stock Center, Department of Biochemistry, The Ohio State University, Columbus, OH, USA. (http://www.bgsc.org/ ) | | | |

**References**

24. Itaya M, Sakaya N, Matsunaga S, Fujita K, Kaneko S (2006) Conjugational transfer kinetics of pLS20 between Bacillus subtilis in liquid medium. Biosci Biotechnol Biochem 70: 740-742. JST.JSTAGE/bbb/70.740 [pii].

27. Singh PK, Ramachandran G, Duran-Alcalde L, Alonso C, Wu LJ, Meijer WJ (2012) Inhibition of Bacillus subtilis natural competence by a native, conjugative plasmid-encoded comK repressor protein. Environ Microbiol 14: 2812-2825. 10.1111/j.1462-2920.2012.02819.x [doi].

60. Zhang XZ, Zhang YH (2011) Simple, fast and high-efficiency transformation system for directed evolution of cellulase in Bacillus subtilis. Microb Biotechnol 4: 98-105. 10.1111/j.1751-7915.2010.00230.x [doi].

68. Bullock WO, Fernandez JM, Short JM (1987) XL1-blue: a high efficiency plasmid transforming *recA* *Escherichia coli* strain with Beta-galactosidase selection. Biotechniques 5: 376-379.

69. Yazgan A, Ozcengiz G, Marahiel MA (2001) Tn10 insertional mutations of Bacillus subtilis that block the biosynthesis of bacilysin. Biochim Biophys Acta 1518: 87-94. S0167-4781(01)00182-8 [pii].

70. Itaya M, Kondo K, Tanaka T (1989) A neomycin resistance gene cassette selectable in a single copy state in the Bacillus subtilis chromosome. Nucleic Acids Res 17: 4410.

71. Guerout-Fleury AM, Frandsen N, Stragier P (1996) Plasmids for ectopic integration in Bacillus subtilis. Gene 180: 57-61.
