# Supplementary material for: Mobility of the Native Bacillus subtilis Conjugative Plasmid pLS20 Is Regulated by Intercellular Signaling
Source: PLoS Genet. 2013 Oct 31;9(10):e1003892. doi: 10.1371/journal.pgen.1003892 (PMC3814332; doi:10.1371/journal.pgen.1003892)
Supplement: Table S5 — Oligonucleotides used in these studies. (DOCX) [file pgen.1003892.s006.docx]

| **Table S5.** Oligonucleotides used | |  |
| --- | --- | --- |
| **Name** | **Sequence**(5´-3´) | **Purpose** |
| Xre20UpHind | ccccAAGCTTTATTTTGCGAGGTGGTATAAGTG | cloning *rco_LS20_* |
| Xre20DnNheI | ccTAGCTAGCGTAAAAAAAGGACTGCACTTAGGCTAG |  |
| Rap20UpSal | ggGTCGACAATAGCTGGAGGGAAGTGTATGTT | cloning *rap_LS20_* |
| Rap20DnNhe | gGCTAGCTCATCCTAACGCTTCTGTTATTCTTTGAATTTGC |  |
| oPKS20 | TATGTAAAAAGGTCATGGCAGGCGAA | Amplification *rco_LS20_* up-stream region |
| oPKS21 | ggggGAATTCAATTATTAAAAATTAGAAATGAATACATG |  |
| oPKS22 | ggggCTGCAGTATACCACCTCGCAAAATAAACCCTG | Amplification *rco_LS20_* down-stream region |
| oPKS23 | TATAGGAATTCCAATCTTTCTTCGCATC |  |
| oPKS38 | AAAAACTACGTCATAATTTTTAAATTGTTC | Amplification *rap_LS20_* up-stream region |
| oPKS39 | ttttCTGCAGTATTCAAAAACAAAACTAGTCC |  |
| oPKS40 | ttttGAGCTCCTAAAGAGCAAATTCAAAGAAT | Amplification *rap_LS20_* down-stream region |
| oPKS41 | ATTTAATTCTTTATCAGCTTAATCACT |  |
| oPKS53N | GTTTGTCACCCAGCTGCCGTTAAGGGGTGT | Amplification *phr_LS20_* up-stream region |
| oPKS54 | ttttCTGCAGAATTTTCTTCATCCTAACGCCTCC |  |
| oPKS55N | ttttGAGCTCTCACGGCTGCAGCTGAGGAGAAGATC | Amplification *phr_LS20_* down-stream region |
| oPKS56 | ACGGAACTCTATATCAGCCAAGTATTCAGA |  |
| oGR85 | ttttGCTAGCGTAAGGATGGGGGAATTTTCTTGCGG | Cloning *rap*I gene |
| oGR86 | ttttGCATGCCTACTTAAAATCGCTGCTGCCGATAGAATCCGGCTGATTTTCGTCAATAA |  |
| 5´- overhang sequences are indicated in lower case and restriction sites are underlined | |  |
