# Supplementary material for: Mobility of the Native Bacillus subtilis Conjugative Plasmid pLS20 Is Regulated by Intercellular Signaling
Source: PLoS Genet. 2013 Oct 31;9(10):e1003892. doi: 10.1371/journal.pgen.1003892 (PMC3814332; doi:10.1371/journal.pgen.1003892)
Supplement: Table S6 — Summary of experimental RNA seq conditions, sequence reads, coverage, and quality score. (DOCX) [file pgen.1003892.s007.docx]

| **Table S6. Summary of Experimental conditions, sequence reads, coverage, and quality score.** | | | | | | | | |
| --- | --- | --- | --- | --- | --- | --- | --- | --- |
| **Experiment** | **genotype** | **Total reads (million)** | **Mapped reads (%)** | **Total reads mapped to pLS20** | **Coverage (fold)** | **UniVec reads (%)** | **Quality score ≥ 30 (%)** | **Quality score ≥ 35 (%)** |
| PKS14 | *amyE*::P_spank_-*rco_LS20_*, pLS20cat | 10.0 | 98.51 | 23,595 | 82 | 0.18 | 93.17 | 85.72 |
| PKS11 | *168,* pLS20cat | 12.1 | 93.88 | 494,109 | 95 | 0.24 | 93.19 | 85.75 |
| 168 |  | 9.9 | 94.54 | 1,262 | 79 | 0.56 | 93.02 | 85.43 |
| GR23 | *amyE*::P_spank_-*rap_LS20_*, pLS20cat | 10.8 | 94.82 | 561,305 | 86 | 0.56 | 93.24 | 85.85 |
